# Longitudinal Functional Recovery Following Primary Total Hip Arthroplasty Utilizing a Contemporary Triple-Taper Femoral Stem

**DOI:** 10.7759/cureus.112803

**Published:** 2026-07-16

**Authors:** Ahmed Siddiqi, Elizabeth Diaz, Elaine Justice, Paul B Jacob

**Affiliations:** 1 Orthopedic Surgery, Texas Center for Joint Replacement, Plano, USA; 2 Orhtopedic Surgery, Texas Health Resources, Dallas, USA; 3 Orthopedic Surgery, Texas Center for Joint Replacement, Dallas, USA; 4 Orthopedic Surgery, Oklahoma Joint Reconstruction Institute, Oklahoma City, USA

**Keywords:** direct anterior tha, insignia, modern tha, total hip arthroplasty (tha), triple taper femoral stem

## Abstract

Background: Contemporary total hip arthroplasty (THA) increasingly emphasizes accelerated recovery, patient-reported outcomes, and value-based care in addition to implant survivorship alone. However, recovery following THA has historically demonstrated substantial variability influenced by demographic, psychosocial, and socioeconomic factors. The purpose of this study was to characterize longitudinal functional recovery trajectories following primary THA utilizing a contemporary triple-taper femoral stem and identify factors associated with early and clinically meaningful postoperative recovery within a standardized contemporary arthroplasty pathway.

Methods: A retrospective review was performed of 1,093 consecutive primary THAs performed between 2022 and 2024 utilizing the Insignia femoral stem through a standardized direct anterior approach and contemporary perioperative recovery pathway. Functional recovery was assessed using the Hip Disability and Osteoarthritis Outcome Score for Joint Replacement (HOOS Jr) collected preoperatively and longitudinally through one year for the entire cohort and through two years for patients eligible at the time of data lock. Recovery trajectory modeling, variability analyses, patient acceptable symptom state (PASS) achievement, substantial clinical benefit (SCB), and multivariable logistic regression analyses evaluating demographic, psychosocial, socioeconomic, and perioperative variables were performed.

Results: Mean HOOS Jr improved from 44.8 ± 7.8 preoperatively to 71.9 ± 9.9 at six weeks, 79.5 ± 8.8 at three months, 86.3 ± 6.9 at one year, and 89.3 ± 6.1 among patients eligible for two-year follow-up (p < 0.001). Longitudinal analyses demonstrated progressive improvement in functional outcomes accompanied by narrowing standard deviation and interquartile ranges over time. Because convergence of scores may partially reflect the bounded nature of the HOOS Jr instrument, these findings should be interpreted alongside the potential influence of ceiling effects. Excellent early functional recovery (HOOS Jr ≥85 at three months) was achieved in 29.3% of patients, while PASS achievement at one year occurred in 85.8% of patients. Minimal clinically important improvement (MCII) and SCB achievement occurred in 95.2% and 100% of patients, respectively. Mental health history, opioid refill utilization, and insurance status demonstrated limited independent influence on longitudinal recovery following multivariable adjustment. Overall complication and reoperation rates remained low.

Conclusion: Primary THA performed within a standardized contemporary arthroplasty pathway demonstrated substantial longitudinal functional improvement and high rates of clinically meaningful recovery. Recovery trajectories became progressively less variable over time, although this observation should be interpreted in light of potential ceiling effects inherent to patient-reported outcome measures. These findings provide a descriptive characterization of recovery following contemporary primary THA but do not isolate the independent contribution of implant design, surgical approach, or perioperative pathway to postoperative recovery.

## Introduction

Total hip arthroplasty (THA) is among the most successful procedures in modern medicine and has historically been evaluated primarily through the lens of implant survivorship, complication avoidance, and radiographic durability [1]. While these metrics remain fundamental, contemporary THA increasingly exists within a substantially different clinical environment characterized by outpatient surgery, accelerated recovery pathways, patient-reported outcome measures (PROMs), and value-based care initiatives. Within this evolving framework, successful THA is no longer defined solely by whether an implant survives, but increasingly by how consistently and efficiently patients recover function following surgery [2,3].

Historically, postoperative recovery following THA has demonstrated substantial heterogeneity [4]. Advanced age, obesity, medical frailty, psychosocial comorbidity, opioid dependence, and socioeconomic disadvantage have each been associated with delayed recovery, inferior patient-reported outcomes, prolonged hospitalization, increased healthcare utilization, and patient dissatisfaction following THA [5]. Consequently, much of the contemporary arthroplasty literature has focused on identifying predictors of inferior outcomes and constructing risk stratification models intended to identify patients at risk for delayed postoperative recovery [6,7].

Simultaneously, contemporary THA has undergone substantial transformation. Standardized perioperative optimization pathways, multimodal analgesia protocols, accelerated mobilization strategies, coordinated discharge planning, advances in surgical technique, and contemporary implant technology have collectively altered the perioperative landscape [8,9]. These developments may not simply improve average postoperative outcomes but may also reduce variability in recovery trajectories across heterogeneous patient populations. In the present study, recovery reproducibility refers to the degree to which postoperative functional recovery trajectories and their variability remain consistent across a heterogeneous patient population over time. From a systems perspective, characterizing recovery reproducibility may therefore represent an increasingly important complement to traditional measures of procedural success.

Reliable recovery following THA depends on multiple factors, including patient optimization, surgical technique, perioperative care, restoration of biomechanics, and durable implant fixation. Contemporary metaphyseal-engaging triple-taper femoral stems were developed to optimize proximal load transfer, multiplanar rotational stability, and biologic fixation while preserving physiologic femoral loading characteristics [10]. Accordingly, evaluation of recovery trajectories in patients treated with contemporary implant designs may provide complementary insights into postoperative functional recovery, although the independent contribution of implant design cannot be determined without a comparator cohort.

Despite these developments, relatively limited literature has characterized longitudinal recovery trajectories following contemporary THA or examined changes in recovery variability throughout postoperative follow-up [11,12]. Improved understanding of recovery reproducibility may enhance patient counseling, expectation setting, and perioperative optimization while providing complementary information beyond traditional measures of implant success.

Therefore, the primary objective of the present study was to characterize longitudinal functional recovery trajectories and changes in recovery variability following primary THA utilizing a contemporary triple-taper femoral stem within a standardized perioperative recovery pathway. The secondary, exploratory objectives were to identify demographic, psychosocial, socioeconomic, and perioperative factors associated with the achievement of excellent early functional recovery and patient acceptable symptom state (PASS). We hypothesized that patients undergoing contemporary primary THA would demonstrate progressive improvement in postoperative function with decreasing variability in recovery trajectories over longitudinal follow-up. Because the Hip Disability and Osteoarthritis Outcome Score for Joint Replacement (HOOS Jr) is a bounded PROM, any observed narrowing of postoperative score dispersion was interpreted in the context of potential ceiling effects rather than assumed to represent true convergence of recovery consistency.

## Materials and methods

Study design and patient selection

Following institutional review board approval (WCG IRB Protocol No. 20262057), a retrospective cohort study was performed of consecutive patients undergoing elective primary total hip arthroplasty (THA) between January 1, 2022, and January 1, 2024, at a single high-volume arthroplasty center. All procedures were performed by a single fellowship-trained adult reconstruction surgeon utilizing a standardized robotic-assisted direct anterior approach and contemporary perioperative recovery pathway.

Patients were identified through the institution's prospectively maintained arthroplasty registry and implant utilization database. Demographic, perioperative, clinical, and patient-reported outcome data were routinely collected as part of standard clinical care and retrospectively extracted for analysis. Inclusion criteria consisted of elective primary THA utilizing the Insignia femoral stem (Stryker Orthopaedics, Mahwah, NJ), a contemporary metaphyseal-engaging triple-taper cementless femoral stem, with a minimum one-year clinical follow-up or documented complication, reoperation, or revision within the first postoperative year. Exclusion criteria included THA performed for acute fracture, revision THA, conversion THA, oncologic reconstruction, active infection, and patients without a minimum one-year follow-up. Because patient enrollment continued through January 1, 2024, not all patients were eligible to reach the two-year follow-up interval at the time of data collection. Accordingly, longitudinal analyses through two years included only patients eligible for that time point.

Surgical technique and perioperative recovery pathway

All procedures were performed utilizing a direct anterior approach on a specialized anterior hip table (HANA table; Mizuho OSI, Union City, CA). Acetabular preparation and component placement were performed using the MAKO SmartRobotics System (Stryker Orthopaedics, Mahwah, NJ). Femoral preparation was performed manually utilizing sequential broaching according to the manufacturer's recommended surgical technique.

A standardized multimodal perioperative recovery pathway emphasizing opioid minimization, accelerated mobilization, early functional restoration, and discharge home was utilized throughout the study period. The pathway included standardized anesthesia protocols, perioperative multimodal analgesia, immediate postoperative weight-bearing as tolerated, same-day physical therapy evaluation, early ambulation, coordinated discharge planning, and standardized postoperative follow-up.

Recovery and clinical outcome assessment

Demographic variables included age, sex, and body mass index (BMI). Medical comorbidity variables included American Society of Anesthesiologists (ASA) classification, diabetes mellitus, chronic kidney disease, hypertension, hyperlipidemia, and smoking history. Psychosocial variables included documented mental health history and postoperative opioid refill utilization. Systems-level variables included insurance type, discharge disposition, home physical therapy utilization, skin-to-skin operative time, and length of stay.

Patient-reported outcomes were prospectively collected as part of the institution's routine arthroplasty registry using the HOOS Jr, a validated six-item instrument scored from 0 to 100, with higher scores indicating superior hip function and health status [13]. Questionnaires were routinely administered preoperatively and at approximately six weeks, three months, six months, one year, and two years following surgery. PROM completion rates were 100% at six weeks, three months, and six months, and 98% at one year. Among the 872 patients eligible for two-year follow-up, 785 (90.0%) completed the two-year assessment.

Longitudinal mixed-effects modeling incorporated all available HOOS Jr observations, allowing patients with incomplete longitudinal follow-up to contribute available outcome data while accounting for repeated within-patient measurements.

The primary study endpoint was longitudinal functional recovery as assessed by serial HOOS Jr scores over time. Excellent early functional recovery, defined as achievement of a HOOS Jr score ≥85 at three months postoperatively, was evaluated as an exploratory secondary endpoint to identify patients demonstrating rapid postoperative recovery. Because no universally accepted early recovery threshold exists for the HOOS Jr at three months, this threshold was selected a priori for exploratory analysis.

Secondary endpoints included longitudinal recovery trajectories, achievement of the PASS, achievement of SCB, complications, readmissions, reoperations, and revision surgery. PASS and SCB thresholds for the HOOS Jr were defined according to previously published threshold values [14].

Statistical analysis

Continuous variables are presented as mean ± standard deviation, while categorical variables are presented as frequencies and percentages.

Longitudinal changes in HOOS Jr scores were evaluated using a linear mixed-effects model with patient included as a random effect to account for repeated measurements over time. Postoperative time was modeled as a fixed effect. An unstructured covariance matrix was specified to account for within-patient correlation across repeated observations, and estimated marginal means were compared using Bonferroni-adjusted pairwise comparisons. Because HOOS Jr is a bounded patient-reported outcome measure, observed reductions in score variability over time were interpreted descriptively and considered alongside the potential influence of ceiling effects rather than assumed to represent true convergence of recovery consistency.

Patients achieving and not achieving excellent early functional recovery (HOOS Jr ≥85 at three months) were initially compared using univariate analyses. Multivariable logistic regression was subsequently performed to identify independent factors associated with achievement of this exploratory secondary endpoint. Variables entered into the multivariable model were selected a priori based on clinical relevance and previously reported predictors of recovery following THA. Covariates included age, sex, BMI, preoperative HOOS Jr, ASA classification, diabetes mellitus, chronic kidney disease, hypertension, hyperlipidemia, smoking history, mental health history, postoperative opioid refill utilization, insurance type, operative time, length of stay, discharge disposition, and home physical therapy utilization.

A separate multivariable logistic regression model was performed to identify independent predictors of PASS achievement at one year. Covariates included age, sex, BMI, ASA classification, Charlson comorbidity index (modeled as a continuous variable), documented depression and/or anxiety, chronic opioid use, preoperative HOOS Jr score (per 10-point increase), and Tönnis grade (grade 3 versus grades 0-2).

Odds ratios (ORs) with corresponding 95% confidence intervals (CIs) were reported. Statistical significance was defined as p < 0.05. All statistical analyses were performed using Statistical Product and Service Solutions (SPSS, version 29.0; IBM SPSS Statistics for Windows, Armonk, NY).

## Results

Patient demographics and perioperative characteristics

A total of 1,093 primary robotic-assisted direct anterior THAs utilizing the Insignia triple-taper cementless femoral stem met the inclusion criteria. Mean follow-up was 23.7 ± 5.6 months. Mean patient age was 67.6 ± 11.6 years, and mean BMI was 29.3 ± 6.6 kg/m². All patients completed a minimum of one year of clinical follow-up. Of the 872 patients eligible to reach the two-year follow-up interval at the time of data collection, 785 (90.0%) completed a two-year patient-reported outcome assessment. Baseline demographic, psychosocial, socioeconomic, and perioperative characteristics are summarized in Table 1.

**Table 1:** Baseline demographic, psychosocial, socioeconomic, and perioperative characteristics Abbreviations: THA, total hip arthroplasty; ASA, American Society of Anesthesiologists

Characteristic	Cohort
Number of THAs	1,093
Mean follow-up, months	23.7 ± 5.6
Age, years	67.6 ± 11.6
Female sex, n (%)	612 (56.0%)
Body mass index, kg/m²	29.3 ± 6.6
Smoking history, n (%)	132 (12.1%)
Diabetes mellitus, n (%)	168 (15.4%)
Hypertension, n (%)	702 (64.2%)
Hyperlipidemia, n (%)	641 (58.6%)
Chronic kidney disease, n (%)	61 (5.6%)
Mental health history, n (%)	284 (26.0%)
Postoperative opioid refill utilization, n (%)	218 (19.9%)
ASA classification 1, n (%)	74 (6.8%)
ASA classification 2, n (%)	547 (50.0%)
ASA classification 3, n (%)	451 (41.3%)
ASA classification 4, n (%)	21 (1.9%)
Medicare insurance, n (%)	514 (47.0%)
Commercial insurance, n (%)	503 (46.0%)
Medicaid insurance, n (%)	49 (4.5%)
Other insurance, n (%)	27 (2.5%)
Operative time, minutes	31.6 ± 4.8
Length of stay, hours	16.1 ± 3.0
Direct discharge home, n (%)	1,051 (96.2%)
Home physical therapy utilization, n (%)	184 (16.8%)
Dual mobility utilization, n (%)	74 (6.8%)
Charlson Comorbidity Index (CCI)	Mean 3.1 ± 1.8 (range 0-9)
CCI 0-2	512 (46.8%)
CCI 3-4	378 (34.6%)
CCI ≥5	203 (18.6%)
Tönnis Grade	
Grade 0-1	98 (9.0%)
Grade 2	487 (44.6%)
Grade 3	508 (46.5%)

All procedures were performed utilizing robotic assistance through a direct anterior approach within a standardized perioperative recovery pathway. Mean operative time was 31.6 ± 4.8 minutes, and mean length of stay was 16.1 ± 3.0 hours. The majority of patients were discharged directly home following surgery.

Longitudinal functional recovery

Substantial and progressive improvement in patient-reported functional outcomes was observed throughout the postoperative period. Mean HOOS Jr improved from 44.8 ± 7.8 preoperatively to 71.9 ± 9.9 at six weeks, 79.5 ± 8.8 at three months, 83.2 ± 8.1 at six months, 86.3 ± 6.9 at one year, and 89.3 ± 6.1 among patients eligible for two-year follow-up (all p < 0.001 versus baseline).

**Figure 1:**
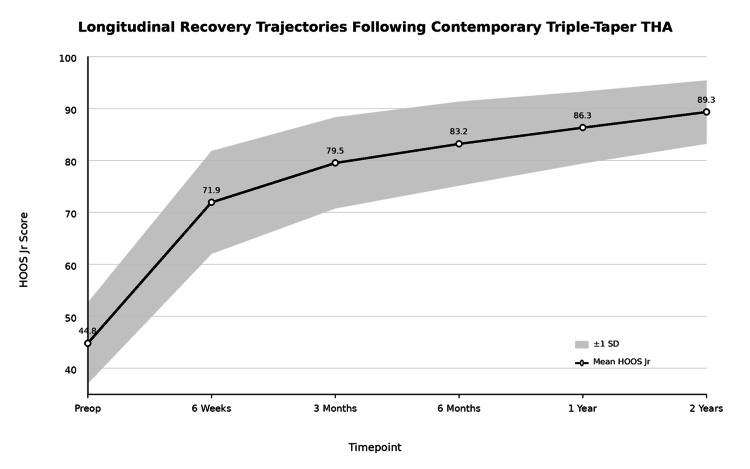
Longitudinal functional recovery following primary total hip arthroplasty Line graph demonstrating longitudinal Hip Disability and Osteoarthritis Outcome Score for Joint Replacement (HOOS Jr) scores following primary total hip arthroplasty in the study cohort. Mean HOOS Jr score improved from 44.8 preoperatively to 71.9 at 6 weeks, 79.5 at 3 months, 83.2 at 6 months, 86.3 at 1 year, and 89.3 among patients eligible for the 2-year follow-up (all p < 0.001 versus baseline). Progressive improvement in postoperative function was observed throughout the follow-up period. The narrowing variability at later postoperative time points should be interpreted in conjunction with the potential influence of ceiling effects as HOOS Jr scores approached the upper limit of the instrument. Abbreviation: HOOS Jr, Hip Disability and Osteoarthritis Outcome Score for Joint Replacement

Linear mixed-effects modeling demonstrated a significant effect of postoperative time on HOOS Jr scores (overall p < 0.001), confirming progressive functional improvement throughout longitudinal follow-up. While modest variability in recovery was observed during the early postoperative period, both the standard deviation and interquartile range of HOOS Jr scores progressively decreased by one and two years postoperatively. Although this finding is consistent with reduced dispersion of postoperative outcomes, it should be interpreted in the context of the potential influence of ceiling effects inherent to bounded patient-reported outcome measures (Figure 1).

**Figure 2:**
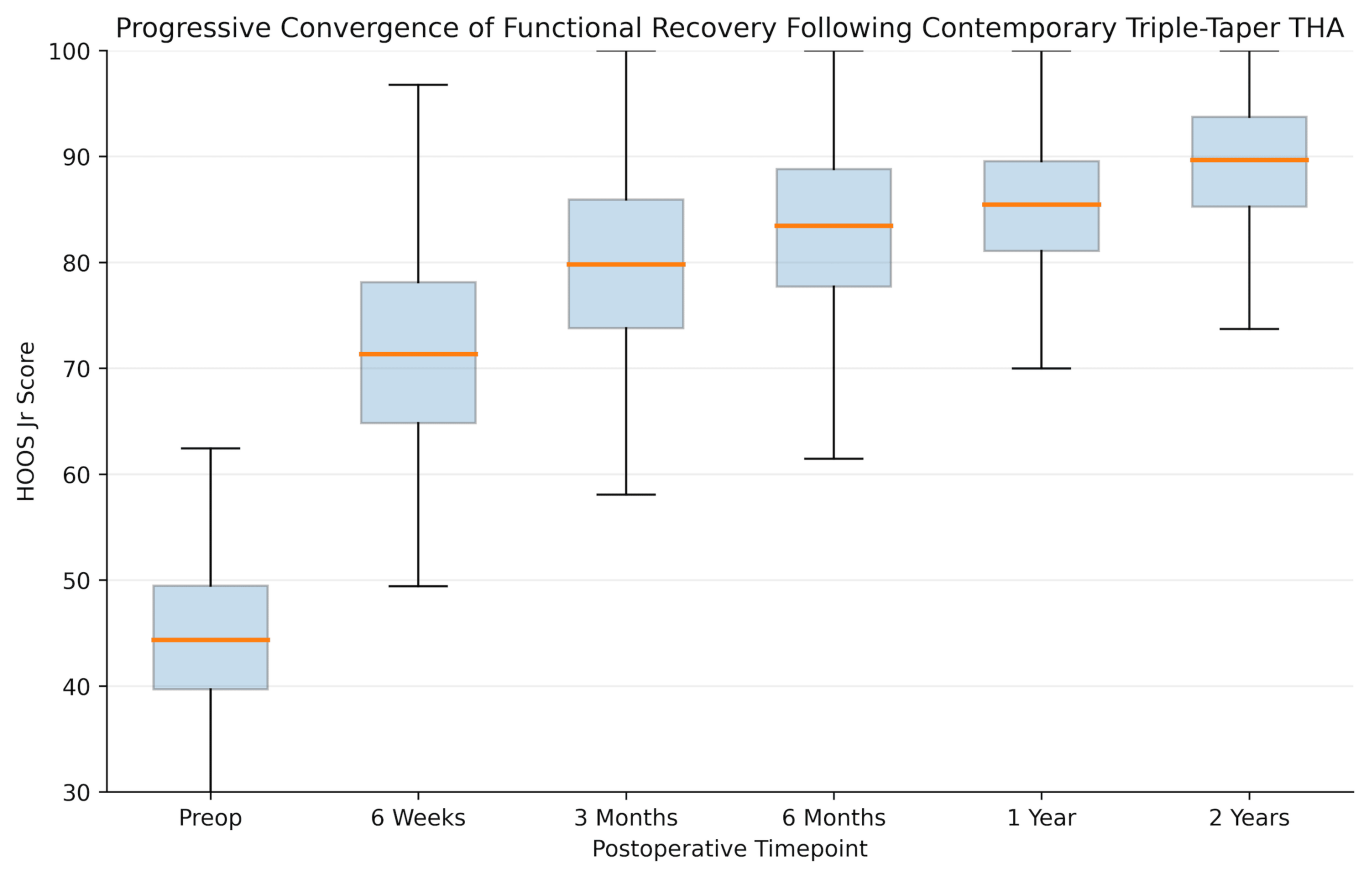
Longitudinal distribution of HOOS Jr scores following primary total hip arthroplasty Box-and-whisker plots demonstrating the distribution of postoperative HOOS Jr scores over longitudinal follow-up following primary total hip arthroplasty in the study cohort. Broader variability was observed during the early postoperative period, whereas the distribution of postoperative scores progressively narrowed at later follow-up. Because HOOS Jr is a bounded patient-reported outcome measure, reduced score dispersion should be interpreted alongside the potential influence of ceiling effects rather than assumed to represent true convergence of recovery trajectories. Two-year data are presented for the 785 patients who completed follow-up among the 872 patients eligible to reach the two-year time point. Abbreviation: HOOS Jr, Hip Disability and Osteoarthritis Outcome Score for Joint Replacement

Interquartile recovery ranges similarly narrowed throughout follow-up, consistent with decreasing dispersion of postoperative HOOS Jr scores across the study cohort. As with the standard deviation, this finding should be interpreted alongside the potential influence of ceiling effects as postoperative scores approached the upper range of the HOOS Jr instrument. Longitudinal recovery trajectory data are summarized in Table 2 and illustrated in Figure 2.

**Table 2:** Longitudinal functional recovery and variability in HOOS Jr scores following primary total hip arthroplasty Progressive improvement in mean HOOS Jr scores was accompanied by decreasing standard deviation and interquartile range over longitudinal follow-up. Because HOOS Jr is a bounded patient-reported outcome measure, reductions in score variability should be interpreted alongside the potential influence of ceiling effects and not assumed to represent true convergence of recovery trajectories. *Two-year values are reported for the 785 patients who completed two-year follow-up among the 872 patients eligible to reach that time point. Abbreviations: HOOS Jr, Hip Disability and Osteoarthritis Outcome Score for Joint Replacement

Postoperative Timepoint	Mean HOOS Jr	Standard Deviation	Interquartile Range
Preoperative	44.8	7.8	39-50
6 weeks	71.9	9.9	65-79
3 months	79.5	8.8	74-86
6 months	83.2	8.1	78-89
1 year	86.3	6.9	82-91
2 years*	89.3	6.1	85-94

Early functional recovery

Excellent early functional recovery, defined as a HOOS Jr score ≥85 at three months postoperatively, was achieved in 320 patients (29.3%). Univariate analysis demonstrated no clinically meaningful differences in age, BMI, smoking history, diabetes mellitus, chronic kidney disease, operative time, or length of stay between patients achieving and not achieving excellent early functional recovery.

Multivariable logistic regression demonstrated no significant independent association between achievement of the exploratory early functional recovery endpoint and age, sex, BMI, diabetes mellitus, chronic kidney disease, hypertension, hyperlipidemia, smoking history, operative time, length of stay, insurance type, discharge disposition, or home physical therapy utilization (all p > 0.05). The ASA class ≥3 demonstrated a trend toward lower rates of excellent early recovery but did not reach statistical significance (OR: 0.76; 95% CI: 0.57-1.01; p = 0.052). Complete multivariable regression results are presented in Table 3.

**Table 3:** Multivariable logistic regression identifying factors associated with excellent early functional recovery Primary outcome: Achievement of the exploratory early functional recovery endpoint (HOOS Jr ≥85 at 3 months postoperatively). Abbreviations: HOOS Jr, Hip Disability and Osteoarthritis Outcome Score for Joint Replacement; ASA, American Society of Anesthesiologists; OR, odds ratio; CI, confidence interval

Covariate	Odds Ratio	95% Confidence Interval	p-value
Age	0.99	0.98-1.01	0.41
Female sex	0.93	0.71-1.21	0.58
Body mass index	0.98	0.96-1.01	0.19
Preoperative HOOS Jr	1.02	0.99-1.04	0.11
Smoking history	0.88	0.59-1.31	0.53
Diabetes mellitus	0.91	0.64-1.31	0.61
Hypertension	0.94	0.70-1.27	0.69
Hyperlipidemia	1.01	0.75-1.35	0.95
Chronic kidney disease	0.79	0.43-1.44	0.44
Mental health history	0.84	0.63-1.12	0.23
Postoperative opioid refill utilization	0.81	0.59-1.11	0.18
ASA classification ≥3	0.76	0.57-1.01	0.052
Medicare insurance	0.92	0.68-1.25	0.59
Medicaid insurance	0.71	0.36-1.39	0.31
Operative time	0.99	0.96-1.02	0.47
Length of stay	0.97	0.91-1.04	0.39
Home physical therapy utilization	0.89	0.62-1.28	0.53
Direct discharge home	1.11	0.61-2.04	0.73

Psychosocial and socioeconomic recovery

Documented mental health history and postoperative opioid refill utilization demonstrated modest associations with delayed early recovery on univariate analysis; however, these associations were attenuated following multivariable adjustment and did not independently predict excellent early functional recovery.

Similarly, insurance-based subgroup analyses demonstrated comparable longitudinal improvement across Medicare, commercial insurance, and other payer groups without clinically meaningful differences in postoperative functional outcomes at the latest follow-up.

**Figure 3:**
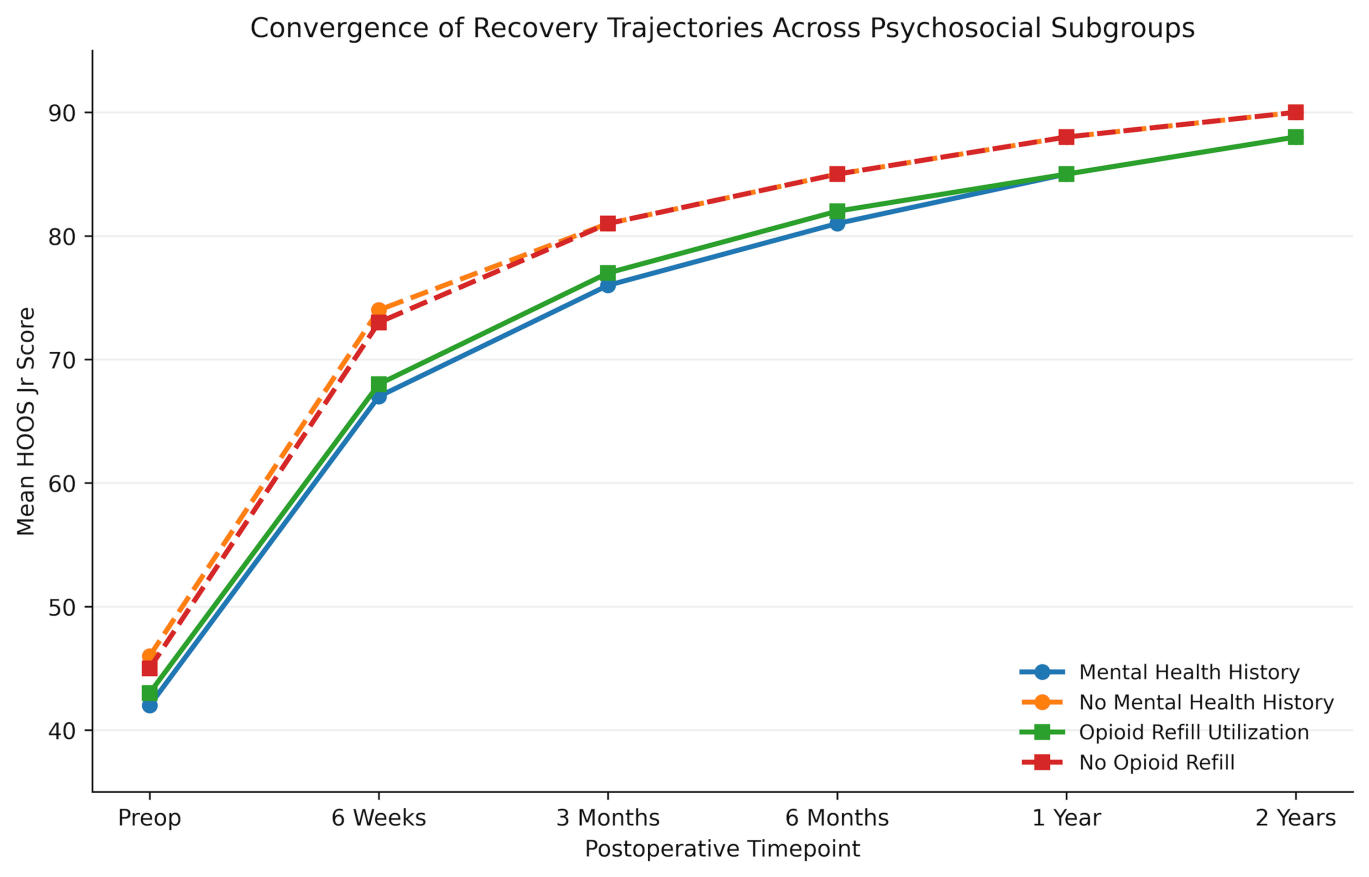
Longitudinal recovery trajectories stratified by psychosocial characteristics Longitudinal recovery curves demonstrating postoperative HOOS Jr trajectories stratified by documented mental health history and postoperative opioid refill utilization following primary total hip arthroplasty in the study cohort. Although modest early differences in postoperative recovery were observed between psychosocial subgroups, substantial improvement in HOOS Jr scores occurred across all groups throughout longitudinal follow-up. The apparent reduction in subgroup differences at later follow-up should be interpreted in the context of the observational study design and the potential influence of ceiling effects inherent to bounded patient-reported outcome measures rather than as evidence that the perioperative pathway or implant independently reduced psychosocial recovery disparities. Abbreviation: HOOS Jr, Hip Disability and Osteoarthritis Outcome Score for Joint Replacement

Longitudinal subgroup recovery curves demonstrated progressive improvement in HOOS Jr scores across mental health history and postoperative opioid refill utilization cohorts throughout follow-up. Although early differences in postoperative recovery were observed between subgroups, these differences became less pronounced over time. Because this was a single-arm observational cohort and HOOS Jr is a bounded patient-reported outcome measure, these findings should be interpreted descriptively and in the context of potential ceiling effects rather than as evidence that the standardized pathway or implant independently reduced psychosocial recovery differences (Figure 3).

PASS and SCB achievement

PASS at one year was achieved in the substantial majority of patients. Multivariable logistic regression demonstrated that higher preoperative HOOS Jr scores were independently associated with achieving PASS (OR: 1.48 per 10-point increase; 95% CI: 1.30-1.68; p < 0.001). Conversely, higher BMI (OR: 0.94 per kg/m²; 95% CI: 0.91-0.97; p = 0.001), greater Charlson comorbidity index (OR: 0.87 per point; 95% CI: 0.78-0.97; p = 0.012), documented depression or anxiety (OR: 0.67; 95% CI: 0.48-0.93; p = 0.018), chronic opioid use (OR: 0.45; 95% CI: 0.27-0.74; p = 0.002), and Tönnis grade 3 osteoarthritis (OR: 0.59; 95% CI: 0.39-0.90; p = 0.014) were independently associated with a lower likelihood of achieving PASS. Increasing age demonstrated a modest association with reduced PASS achievement (OR: 0.98 per year; 95% CI: 0.96-1.00; p = 0.047), whereas sex and ASA classification were not independently associated with PASS achievement (Table 4).

**Table 4:** Multivariable logistic regression identifying independent predictors of achieving patient acceptable symptom state (PASS) at one year Model performance: Area under the receiver operating characteristic curve (AUC), 0.79; Nagelkerke R², 0.33. Odds ratios for continuous variables represent the change in odds associated with the unit increase specified for each variable. Preoperative HOOS Jr was modeled per 10-point increase. Abbreviations: OR, odds ratio; CI, confidence interval; PASS, Patient Acceptable Symptom State; HOOS Jr, Hip Disability and Osteoarthritis Outcome Score for Joint Replacement; BMI, body mass index; ASA, American Society of Anesthesiologists

Variable	Odds Ratio	95% CI	p-value
Age (per year)	0.98	0.96-1.00	0.047
Body mass index (per kg/m²)	0.94	0.91-0.97	0.001
Female sex	1.09	0.78-1.52	0.612
ASA classification ≥3	0.74	0.54-1.01	0.058
Charlson comorbidity index (per point)	0.87	0.78-0.97	0.012
Documented depression or anxiety	0.67	0.48-0.93	0.018
Chronic opioid use	0.45	0.27-0.74	0.002
Preoperative HOOS Jr (per 10-point increase)	1.48	1.30-1.68	<0.001
Tönnis grade 3	0.59	0.39-0.90	0.014

**Figure 4:**
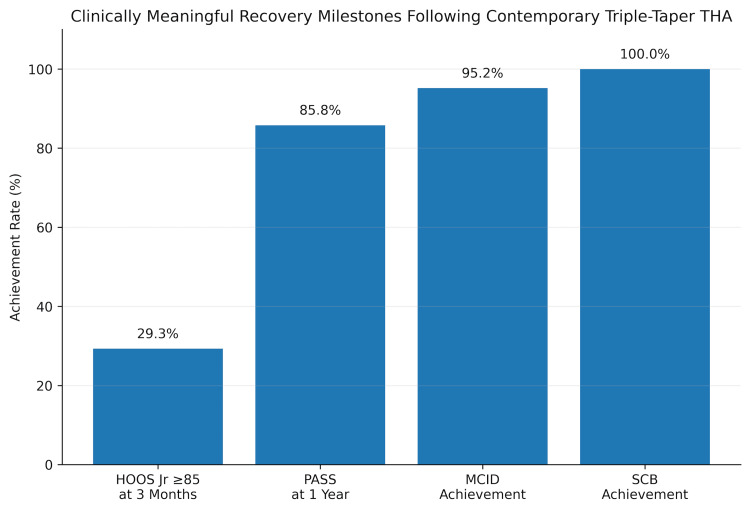
Clinically meaningful recovery outcomes following primary total hip arthroplasty Bar graph demonstrating the proportion of patients achieving clinically meaningful recovery outcomes following primary total hip arthroplasty in the study cohort. Excellent early functional recovery (HOOS Jr ≥85 at 3 months) was achieved in 29.3% of patients, whereas Patient Acceptable Symptom State (PASS) at 1 year, Minimal Clinically Important Improvement (MCII), and Substantial Clinical Benefit (SCB) were achieved in 85.8%, 95.2%, and 100% of patients, respectively. These findings demonstrate high rates of clinically meaningful improvement across multiple validated patient-reported outcome thresholds. Abbreviations: HOOS Jr, Hip Disability and Osteoarthritis Outcome Score for Joint Replacement; PASS, Patient Acceptable Symptom State; MCII, Minimal Clinically Important Improvement; SCB, Substantial Clinical Benefit

SCB was achieved in all patients and was therefore summarized descriptively rather than modeled using multivariable regression because of the absence of outcome variability. Clinically meaningful recovery outcomes, including excellent early functional recovery, PASS, MCII, and SCB achievement rates, are summarized in Table 5 and illustrated in Figure 4.

**Table 5:** Clinically meaningful recovery outcomes following primary total hip arthroplasty Clinically meaningful functional improvement was achieved in the substantial majority of patients across multiple validated outcome thresholds. SCB was achieved in all patients, whereas MCII and PASS were achieved in 95.2% and 85.8% of patients, respectively. Abbreviations: HOOS Jr, Hip Disability and Osteoarthritis Outcome Score for Joint Replacement; PASS, Patient Acceptable Symptom State; SCB, Substantial Clinical Benefit; MCII, Minimal Clinically Important Improvement

Outcome Measure	Achievement Rate
Excellent early functional recovery (HOOS Jr ≥85 at 3 months)	320/1,093 (29.3%)
PASS achievement at 1 year	938/1,093 (85.8%)
SCB achievement	1,093/1,093 (100%)
Minimal Clinically Important Improvement (MCII) achievement	1,041/1,093 (95.2%)

Complications and reoperations

No intraoperative periprosthetic femoral fractures occurred during primary THA. Overall complication and reoperation rates remained low throughout the study period. Five patients (0.46%) underwent reoperation, including one postoperative Vancouver B2 periprosthetic femoral fracture requiring femoral component revision, two Vancouver C periprosthetic femoral fractures treated with open reduction and internal fixation, and two early postoperative periprosthetic joint infections successfully managed with debridement, antibiotics, and implant retention.

No postoperative dislocations, aseptic femoral loosening, or deaths occurred during the study period.

## Discussion

The principal finding of the present study was not simply that patients experienced substantial improvement following primary THA, but that postoperative functional recovery demonstrated progressively less dispersion in patient-reported outcomes over longitudinal follow-up despite considerable baseline demographic, medical, psychosocial, and socioeconomic heterogeneity. Although contemporary THA literature has largely focused on identifying predictors of inferior outcomes, the present investigation approached recovery from a different perspective by characterizing longitudinal recovery trajectories and evaluating changes in recovery variability over time. Progressive improvement in HOOS Jr scores, high rates of clinically meaningful recovery, and decreasing dispersion across serial postoperative assessments demonstrate that contemporary primary THA performed within a standardized perioperative pathway can achieve excellent functional outcomes across a diverse patient population.

Traditionally, outcomes following THA have been defined by implant survivorship, complication rates, radiographic fixation, and revision avoidance [10,15-17]. While these metrics remain fundamental, modern arthroplasty increasingly emphasizes outpatient surgery, enhanced recovery protocols, patient-reported outcome measures, and value-based care. Within this framework, consistency of recovery represents a potentially important complement to traditional outcome measures. Two patients may ultimately achieve similar postoperative function while experiencing markedly different recovery trajectories. Therefore, evaluating not only the magnitude but also the variability of recovery may provide additional insight into the reliability of contemporary arthroplasty care.

The longitudinal analyses support this concept. Mean HOOS Jr scores improved significantly throughout follow-up, while both the standard deviation and interquartile range of postoperative scores progressively decreased over time. Because HOOS Jr is a bounded patient-reported outcome measure with an upper limit of 100, some reduction in score variability is expected as postoperative scores approach the ceiling of the instrument. Consequently, the observed narrowing of score dispersion should not be interpreted as definitive evidence of true recovery convergence. Nevertheless, the persistence of reduced interquartile variability alongside uniformly high rates of clinically meaningful improvement suggests that contemporary primary THA can achieve consistently favorable functional outcomes across a heterogeneous patient population. Previous investigations have largely emphasized average improvements in patient-reported outcomes following THA [18,19]; comparatively fewer studies have examined longitudinal variability in recovery itself. From a health systems perspective, understanding recovery variability may complement traditional outcome assessment and inform patient counseling, perioperative optimization, resource utilization, and value-based care initiatives.

An additional notable finding was the limited independent influence of several traditionally recognized recovery modifiers. Mental health history, postoperative opioid refill utilization, and insurance status demonstrated modest associations with delayed early recovery on univariate analysis, but these relationships were attenuated following multivariable adjustment. Numerous studies have demonstrated that depression, anxiety, pain catastrophizing, opioid dependence, and socioeconomic disadvantage are associated with inferior patient-reported outcomes and delayed recovery following THA and TKA [20-28]. Similarly, obesity, medical comorbidity, and incomplete preoperative optimization have consistently been associated with increased perioperative complications, healthcare utilization, and inferior outcomes following THA, underscoring the importance of patient optimization before surgery [29,30]. Our findings should not be interpreted as suggesting that these factors are unimportant. Rather, they suggest that favorable functional recovery can be achieved across patients with diverse baseline characteristics within a standardized contemporary arthroplasty program, although these observations should not be interpreted as evidence that the perioperative pathway independently mitigated the effects of these risk factors.

Importantly, determinants of rapid early recovery differed from those associated with longer-term patient satisfaction. Few variables independently predicted achievement of the exploratory early functional recovery endpoint at three months, whereas higher preoperative HOOS Jr scores increased the likelihood of achieving the PASS at one year. Conversely, increasing BMI, greater comorbidity burden, depression or anxiety, chronic opioid use, advanced radiographic osteoarthritis, and increasing age were independently associated with lower odds of achieving PASS. These findings suggest that factors influencing the pace of early recovery differ from those determining whether patients ultimately perceive their postoperative function as acceptable, highlighting the importance of evaluating both longitudinal recovery trajectories and clinically meaningful recovery thresholds.

The present findings should be interpreted within the context of the standardized surgical technique, perioperative pathway, and implant utilized throughout the study. All procedures were performed using a robotic-assisted direct anterior approach, a contemporary perioperative recovery pathway, and the same metaphyseal-engaging triple-taper cementless femoral stem. This uniform approach minimized variation in surgical technique and implant selection within the cohort, providing a consistent clinical setting in which to characterize postoperative recovery. Because no comparator cohort was included, the present study cannot determine the independent contribution of implant design, surgical approach, robotic assistance, or perioperative management to the observed outcomes. Rather, these findings demonstrate that excellent functional recovery, high rates of clinically meaningful improvement, and a low complication burden were observed within this contemporary treatment strategy.

The high rates of MCII, PASS, and SCB further reinforce the favorable functional outcomes observed in this cohort. While the exploratory threshold of HOOS Jr ≥85 at three months identified patients demonstrating particularly rapid early recovery, clinically meaningful improvement and acceptable symptom state were ultimately achieved by the substantial majority of patients. These findings emphasize that, although recovery velocity varies during the early postoperative period, long-term functional restoration remains highly favorable following contemporary primary THA.

This study has several limitations. First, its retrospective design introduces the potential for selection bias and unmeasured confounding. Second, all procedures were performed by a single high-volume fellowship-trained arthroplasty surgeon utilizing a standardized perioperative pathway, which may limit generalizability to other practice settings. Third, the absence of a comparator cohort precludes conclusions regarding the independent effect of implant design, surgical approach, robotic assistance, or perioperative management on postoperative recovery. Fourth, although longitudinal patient-reported outcome follow-up was excellent, these measures remain susceptible to ceiling effects during longer-term follow-up, and some reduction in score variability likely reflects this statistical phenomenon rather than true convergence of recovery trajectories. Finally, despite multivariable adjustment, residual confounding from unmeasured clinical, psychosocial, or socioeconomic variables cannot be excluded.

## Conclusions

Contemporary primary THA performed within a standardized robotic-assisted direct anterior approach and a contemporary perioperative recovery pathway was associated with substantial functional improvement, high rates of clinically meaningful recovery, and a low complication burden over two years of follow-up. Longitudinal analyses demonstrated progressive improvement in patient-reported outcomes accompanied by decreasing dispersion of HOOS Jr scores over time, although these findings should be interpreted in the context of potential ceiling effects inherent to patient-reported outcome measures. Collectively, these results demonstrate that excellent functional outcomes can be achieved across a diverse patient population within a standardized contemporary arthroplasty program. Further comparative studies are warranted to determine the independent contributions of implant design, surgical technique, robotic assistance, and perioperative optimization to postoperative recovery following THA.
